# Projecting the chronic disease burden among the adult population in the United States using a multi-state population model

**DOI:** 10.3389/fpubh.2022.1082183

**Published:** 2023-01-13

**Authors:** John P. Ansah, Chi-Tsun Chiu

**Affiliations:** ^1^Center for Community Health Integration, Case Western Reserve University, Cleveland, OH, United States; ^2^Institute of European and American Studies, Academia Sinica, New Taipei, Taiwan

**Keywords:** chronic disease, adult population, multi-state population projection, United States of America, projections

## Abstract

**Introduction:**

As the United States population ages, the adult population with chronic diseases is expected to increase. Exploring credible, evidence-based projections of the future burden of chronic diseases is fundamental to understanding the likely impact of established and emerging interventions on the incidence and prevalence of chronic disease. Projections of chronic disease often involve cross-sectional data that fails to account for the transition of individuals across different health states. Thus, this research aims to address this gap by projecting the number of adult Americans with chronic disease based on empirically estimated age, gender, and race-specific transition rates across predetermined health states.

**Methods:**

We developed a multi-state population model that disaggregates the adult population in the United States into three health states, i.e., (a) healthy, (b) one chronic condition, and (c) multimorbidity. Data from the 1998 to 2018 Health and Retirement Study was used to estimate age, gender, and race-specific transition rates across the three health states, as input to the multi-state population model to project future chronic disease burden.

**Results:**

The number of people in the United States aged 50 years and older will increase by 61.11% from 137.25 million in 2020 to 221.13 million in 2050. Of the population 50 years and older, the number with at least one chronic disease is estimated to increase by 99.5% from 71.522 million in 2020 to 142.66 million by 2050. At the same time, those with multimorbidity are projected to increase 91.16% from 7.8304 million in 2020 to 14.968 million in 2050. By race by 2050, 64.6% of non-Hispanic whites will likely have one or more chronic conditions, while for non-Hispanic black, 61.47%, and Hispanic and other races 64.5%.

**Conclusion:**

The evidence-based projections provide the foundation for policymakers to explore the impact of interventions on targeted population groups and plan for the health workforce required to provide adequate care for current and future individuals with chronic diseases.

## What is already known about this topic?

- Adults population in the United States with chronic diseases is expected to increase.

## What is added by this research?

- An evidence-based age, gender, and race-specific projections of the burden of chronic diseases show that the majority of the adult population 50 years and older, across all races, will have at least one chronic disease by 2050, with the majority between the ages of 60 to 79 years.

## What are the implications for public health?

- The importance of prioritizing the promotion of access to high-quality primary care to provide whole-person care that ensures prevention and management of chronic disease care and addresses evidence-based social determinants of health that increase the risk of developing chronic diseases.

## Introduction

According to the US Centers for Disease Control and Prevention, in 2019, 54.1 million US adults were 65 years or older, representing 16% of the population. By 2040, it is estimated that the number of older adults 65 years and older is expected to reach 80.8 million and 94.7 million by 2060, representing 25% of the US population. An aging population is characterized by the co-occurrence of more than one chronic condition, which is referred to as multimorbidity (1–4).

A meta-analysis of the prevalence of multimorbidity in high, low, and middle-income countries found an overall pooled prevalence of 33.1% (30.0–36.3). There was a considerable difference in the pooled estimates between high-income countries and low and middle-income countries, with prevalence ranging between 37.9 (32.5–43.5) and 29.7% (26.4–33.0), respectively (5). In the United States, data from the 2018 National Health Interview Survey (NHIS) indicates that 27.2% of US adults had multiple chronic conditions. While multimorbidity is not new, there is greater recognition of its impact and the importance of improving outcomes for individuals affected. Multimorbidity is associated with increased mortality (6), reduced quality of life, and functional status (2, 7, 8), increased health services use (3, 9), and higher cost of care.

To better understand the future chronic disease burden, as well as explore the effectiveness of various interventions on the incidence and prevalence of chronic disease, including quality of life outcomes for people with chronic disease, requires an evidence-based and credible forecast of the current and a future number of American adults with chronic disease. Projections of chronic disease often involve cross-sectional data that fails to account for the transition of individuals across different health states. Thus, this research aims to address this gap by projecting the number of adult Americans with chronic disease based on empirically estimated age, gender, and race-specific transition rates across predetermined health states. The evidence-based projections from this research could help healthcare providers to implement interventions for targeted population groups to prevent and or manage their chronic disease and plan for the health workforce required to provide adequate care for current and future individuals with chronic diseases to achieve the quadruple aim of healthcare, i.e., improve population health, reduce cost, and increase patients' and providers satisfaction (10, 11).

## Methods

### Model design

To project the number of Americans 50 years and older with one or more chronic conditions, we developed and validated a dynamic multi-state population model (12–15) to simulate the population of the United States and track their transition to and from three health states. The health states are (a) healthy (adults with no chronic condition), (b) one chronic condition (adult with any one of the nine chronic conditions indicated in the Health and Retirement Survey), and (c) multimorbidity (adults with at least two chronic conditions indicated in the Health and Retirement Survey). For each health state, adult individuals were further divided into a three-dimensional vector: age (from age 50–100 and older), gender (male and female), and race (non-Hispanic white, non-Hispanic black, Hispanic, and other races). To ensure consistency and validation of the model output, an additional state that accounts for the population below 50 years was included to ensure that individuals aged 50 transitions to the adult population's health states. To ensure a consistent aging process, the population aged 50 years and younger was subdivided by age (age 0–age 49). The number of people below age 50 increases by births and net migration (estimated by calibration) and decreases by deaths and becoming age 50. Births were estimated using race-specific fertility rates from the National Vital Statistics report and the fecund female population age 15–49, while life tables informed deaths (16). At the end of each year, the surviving population in each age cohort flows to the subsequent cohort, except the final age cohort, age 100 and older. Transition across health states was determined by 1-year age-gender-race specific transition rates.

#### Health states

The chronic conditions in the Health and Retirement Survey record self-reported lifetime histories of a modest number of illnesses and conditions that are very important to older persons and account for much of the morbidity and mortality among older persons in western societies. The conditions consist of: (a) hypertension, (b) diabetes mellitus, (c) cancer (various types at all bodily sites, except minor skin cancers), (d) chronic lung diseases (often including emphysema, but not asthma), (e) coronary health disease, (f) congestive health failure, (g) stroke (cerebrovascular disease), (h) arthritis (a collection of heterogeneous diseases and Musculoskeletal pain syndromes), and (i) psychiatric problem (in general, not further defined or categorized, except major depressive, depressive symptoms, and dementia). Adult individuals who reported no presence of any chronic conditions were classified as healthy; those who reported only one of the chronic conditions were classified as adults with a single chronic condition, whereas those with at least two or more chronic conditions were classified as adults with multimorbidity.

#### Model assumption

Constant age-gender-race-specific mortality rates were used for the population 50 years and younger. For the adult population, the 1000 bootstrap estimates for all the transitions across the health states accounted for future improvement or deterioration. A race-specific fertility rate was used, while we assumed a constant fertility rate from 2018 over the simulation time. This assumption was deemed appropriate because a fertility rate change will not impact the adult population by 2060. The net migration rate, estimated *via* calibration, was assumed to be constant over the simulation time.

#### Estimation of transition rates

The 1998 to 2018 Health and Retirement Study data (17) was used to estimate the transition rates across health states. The Health and Retirement Study is a longitudinal panel study that surveys a representative sample of more than 26,000 Americans over the age of 50 every two years. The study explores changes in labor force participation and the health transitions that individuals undergo toward the end of their work lives. Since its inception, the study has collected information about income, work, assets, pension plans, health insurance, disability, physical health and functioning, cognitive impairment, and healthcare expenditure.

The input data to the transition rate estimate is in an extended format, and each observation occupies one line of data. Each line of observation includes the individual's age and values of covariates in the model. Everyone has multiple lines of observation. Since the Health and Retirement Study is not an annual survey, we fill in gaps with pseudo-data representing successive years to obtain annual transition probabilities (18). If starting and ending states of an interval are the same, the filled-in data assume that states. If an interval's starting and ending states differ, the filled-in data assume one transition at a random time. Multinomial logistic regressions are then fitted to estimate the probability of transitioning from a starting health state to one of ending health states (including death). Multinomial logistic regression models estimate age, gender, and race-specific transition rates.

### Model validation

The model structure has been validated and used for several publications on similar chronic conditions in other countries (13–15). Thus, the structure of the model has been presented to researchers familiar with chronic disease care in several countries to verify the conceptual framework of the model and its assumptions regarding causal relationships as indicated in the literature cited (19–21). The model structure is grounded in evidence of how individuals transition from a healthy state to a single and multiple chronic conditions over their lifetime. To ensure that the model output is consistent with available data, selected simulated outcomes were compared with available data. The results suggest that the simulated model outputs compare favorably with the available data, demonstrating that the model performs credibly.

## Results

### Transition rates by age, gender, and race

**Figure 1:**
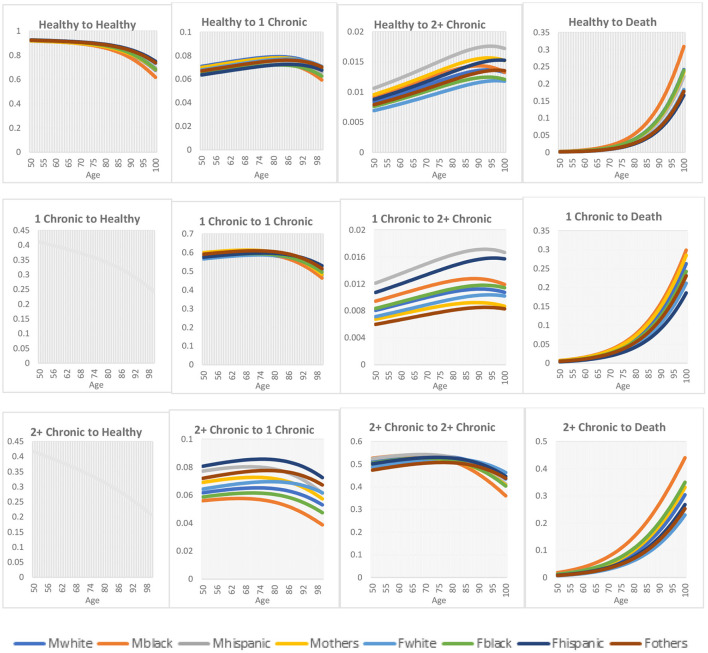
Transition rates across health states. Mwhite: is male non-Hispanic white; Mblack: is male non-Hispanic black; Mhispanic: is male Hispanic; and Mother: is male other races; while Fwhite: is female non-Hispanic white; Fblack: is female non-Hispanic black; Fhispanic: is female Hispanic; and Fothers: is female other races.

Figure 1 shows the age, gender, and race-specific transition rates across the three health states and death. For both gender and race, the progression to worse health status (healthy to one chronic condition, healthy to multimorbidity, and from one chronic condition to multimorbidity) increases with age, except for the progression from healthy to one chronic condition where the transition rates begin to decrease significantly from age 90. Also, mortality across all the health states increases with age. On the contrary, for both gender and race, the regression to a better health status from multimorbidity to one chronic condition decreases with age.

For gender differences, males have a higher rate of progression to a worse health state compared to females, while regression to a better health state was better for females than males. Also, females are more likely to maintain their health status than males. In the case of mortality, males have higher death rates than females. For race differences, non-Hispanic White had a higher transition rate while non-Hispanic Black had the lowest transition rate from a healthy to one chronic condition. For individuals transitioning from healthy to multimorbidity, Hispanics had a higher transition rate for all races, while non-Hispanic Whites had the lowest transition rate. Likewise, Hispanics had the highest transition rates for individuals transitioning from one chronic condition to multimorbidity, while other races had the lowest transition rates. Hispanics had the highest transition rates for regression from multimorbidity to one chronic condition, whereas non-Hispanic Blacks had the lowest transition rates. The transition rates from healthy to death and one chronic condition to death show that non-Hispanic Blacks have the highest transition rates among all the races, whereas Hispanics have the lowest transition rates. For the transition from multimorbidity to death, non-Hispanic Blacks have the highest transition rates, while non-Hispanic Whites have the lowest transition rates.

The results in Table 1 suggest that the number of people in the United States aged 50 years and older will increase by 61.11% (100% confidence interval 57.2%−66.2%) from 137.25 million (135.64–139.18) in 2020 to 221.13 million (213.24–231.34) in 2050. Remarkably, the number of people aged 80 years and older will increase by 137.26% (116.0%−164.6%), from 16.935 million (16.148–17.863) in 2020 to 40.181 million (34.881–47.272) in 2050. Of the population 50 years and older, the number with at least one chronic disease is estimated to increase by 99.5% (95.1%−107.9%) from 71.522 million (69.065–73.781) in 2020 to 142.66 million (134.74–153.39) by 2050. At the same time, those with multimorbidity are projected to increase 91.16% (79.09%−103.24%) from 7.8304 million (6.5965–9.4853) in 2020 to 14.968 million (11.813–19.277) in 2050. The analysis suggests that by 2035, 35.66% (33.36–36.04) of the adult population 50 years and older will have at least one chronic condition, which is expected to increase to 47.81% (46.09–49.71) by 2050. At the same time, 3.659% (2.905–4.696) of the adult population is expected to have multimorbidity, increasing to 5.017% (3.948–6.481) by 2050. Most individuals with at least one chronic condition (62.75% in 2020 and 58.54% in 2050) or multimorbidity (62.9% in 2020 and 58.9% in 2050) are between the ages of 60 to 79 years. However, individuals aged 80 years and older with one chronic condition and multimorbidity are projected to have the highest increase (244.3% for one chronic condition and 202.7% for multimorbidity) from 2020 to 2050.

**Table 1:** Projected number of adults 50 years and older with chronic disease in the United States.

**Age cohort**	**2020**	**2035**	**2050**	**Relative change between 2020 and 2050 (%)**
**Population (million)**				
Total adult population	137.25 [135.64–139.18]	180.40 [175.17–186.85]	221.13 [213.24–231.34]	61.11% [57.21% to 66.22%]
50–59 years	48.318 [48.212–48.419]	55.755 [55.634–55.870]	68.555 [68.401–68.700]	41.88% [41.88% to 41.89%]
60–79 years	72.001 [71.212–72.910]	94.610 [92.326–97.129]	112.40 [109.69–115.39]	56.11% [54.04% to 58.27%]
80 and older	16.935 [16.148–17.863]	30.042 [26.998–33.875]	40.181 [34.881–47.272]	137.267% [116.0% to 164.6%]
≥**1 chronic condition (million)**				
Adult population	71.522 [69.065–73.781]	114.48 [108.87–121.36]	142.66 [134.74–153.39]	99.5% [95.1% to 107.9%]
50–59 years	15.929 [15.181–16.460]	17.688 [16.820–18.305]	22.277 [21.192–23.047]	39.85% [39.59% to 40.01%]
60–79 years	44.881 [43.462–46.090]	70.752 [67.520–73.550]	83.511 [79.670–86.834]	86.07% [83.31% to 88.40%]
80 and older	10.711 [10.012–11.662]	26.040 [23.190–29.936]	36.875 [31.757–44.038]	244.3% [217.2% to 277.6%]
**Multimorbidity (million)**				
Adult population	7.8304 [6.5965–9.4853]	12.085 [9.6091–15.490]	14.968 [11.813–19.277]	91.16% [79.09% to 103.24%]
50–59 years	1.6234 [1.3501–1.9254]	1.7968 [1.4913–2.1314]	2.2662 [1.8802–2.6889]	39.6% [39.26% to 39.66%]
60–79 years	4.9258 [4.1510–6.0167]	7.4836 [5.8964–9.7171]	8.8249 [6.9446–11.454]	79.16% [67.30% to 90.39%]
80 and older	1.2812 [0.9838–1.5817]	2.8050 [1.9896–3.8781]	3.8777 [2.6510–5.5552]	202.7% [169.5% to 251.2%]
Prevalence of ≥1 chronic condition (%)	21.77 [21.05–22.37]	34.66 [33.36–36.04]	47.81 [46.09–49.71]	119.7% [118.9% to 122.2%]
Fraction female	52.57 [51.77–53.20]	53.37 [52.43–54.62]	53.22 [52.07–54.88]	1.25% [0.57% to 3.16%]
Fraction male	47.43 [46.80–48.23]	46.63 [45.38–47.57]	46.78 [45.12–47.93]	−1.39% [−3.59 to −0.61%]
Prevalence of multimorbidity (%)	2.383 [2.007–2.887]	3.659% [2.905–4.696]	5.017 [3.948–6.481]	110.5% [96.7% to 124.5%]
Fraction female	48.33 [44.71–52.14]	49.40 [44.54–55.21]	49.29 [44.21–55.57]	1.98% [−1.10% to 6.59%]
Fraction male	51.67 [47.86–55.29]	50.60 [44.79–55.46]	50.71 [44.43–55.79]	−1.86% [−7.18% to 0.89%]

For gender differences, by 2050, 53.33% (52.07–54.88) of the individuals with one chronic condition are projected to be females, while males form the majority of those with multimorbidity with an estimated 50.71% (44.43–55.79) by 2050.

Tables 2–5 show the race-specific projections. The number of non-Hispanic Whites adults 50 years and older with at least one chronic condition is projected to increase from 46.6159 million (44.786–48.339) in 2020 to 93.026 million (86.516–99.839) by 2050, representing an increase of 99.55% (93.2%−106.5%). Most non-Hispanic Whites with one chronic condition are females between the ages of 60 to 79 years. In addition, the age group with the highest increase in one chronic condition is individuals 80 years and older. Similarly, the number of non-Hispanic Whites with multimorbidity is estimated to increase from 4.8927 million (4.0826–5.9523) in 2020 to 9.12 million (6.9983–12.6401) by 2050. For multimorbidity among the non-Hispanic Whites, the majority are males between the ages of 60 to 79 years.

**Table 2:** Projected number of non-Hispanic White adults 50 years and older with chronic disease (in millions by sex and age) in the United States.

**Age cohort**	**2020**	**2035**	**2050**	**Relative change between 2020 and 2050 (%)**
≥**1 chronic condition (million)**				
50–59 years	10.347 [9.8407–10.739]	11.483 [10.893–11.942]	14.464 [13.725–15.038]	39.79% [39.48%−40.03%]
60–79 years	29.260 [28.395–30.063]	46.139 [44.027–47.866]	54.453 [51.942–56.510]	86.10% [82.93%−87.97%]
80 and older	7.0089 [6.5512–7.5374]	17.040 [15.209–19.300]	24.109 [20.849–28.291]	244.0% [218.3%−275.4%]
Total	46.6159 [44.786–48.339]	74.662 [70.129–79.108]	93.026 [86.516–99.839]	99.55% [93.2%−106.5%]
**Female**				
50–59 years	5.1688 [4.8547–5.4175]	5.7010 [5.3377–5.9886]	7.0841 [6.6341–7.4400]	37.05% [36.65%−37.33%]
60–79 years	15.1119 [14.500–15.652]	24.070 [22.700–25.213]	28.092 [26.472–29.445]	85.89% [82.56%−88.12%]
80 and older	4.2173 [3.8823–4.6583]	10.054 [8.7272–11.916]	14.308 [11.917–17.784]	239.3% [207.0%−281.8%]
**Male**				
50–59 years	5.1783 [4.9860–5.3623]	5.7828 [5.5556–6.0003]	7.3799 [7.0912–7.6559]	42.52% [42.22%−42.77%]
60–79 years	14.148 [13.862–14.451]	22.068 [21.327–22.789]	26.360 [25.470–27.223]	86.32% [83.74%−88.38%]
80 and older	2.7916 [2.6611–2.9202]	6.9860 [6.4725–7.5923]	9.8011 [8.9301–10.897]	251.1% [235.6%−273.2%]
**Multimorbidity (million)**				
50–59 years	0.9972 [0.8259–1.1720]	1.1019 [0.9096–1.2939]	1.3900 [1.1474–1.6326]	39.39% [38.93%−39.31%]
60–79 years	3.0740 [2.6021–3.7479]	4.6747 [3.6213–6.1113]	5.5100 [4.2812–7.1998]	79.24% [64.53%−92.10%]
80 and older	0.8215 [0.6546–1.0324]	1.8122 [1.2028–2.5807]	2.5120 [1.5697–3.8077]	205.8% [139.8%−268.8%]
Total	4.8927 [4.0826–5.9523]	7.5888 [5.7337–9.9859]	9.412 [6.9983–12.6401]	92.368% [71.4%−112.4%]
**Female**				
50–59 years	0.4511 [0.3521–0.5682]	0.4962 [0.3860–0.6250]	0.6167 [0.4795–0.7779]	36.70% [36.17%−36.91%]
60–79 years	1.4434 [1.1306–1.9062]	2.2337 [1.5682–3.2361]	2.6012 [1.8290–3.7738]	80.21% [61.77%−97.98%]
80 and older	0.4669 [0.3514–0.6284]	1.0161 [0.5919–1.6156]	1.4210 [0.7573–2.3860]	204.3% [115.5%−279.7%]
**Male**				
50–59 years	0.5461 [0.4727–0.6140]	0.6058 [0.5224–0.6831]	0.7734 [0.6667–0.8722]	41.61% [41.04%−42.05%]
60–79 years	1.6306 [1.4566–1.8417]	2.4410 [2.0531–2.8751]	2.9088 [2.4522–3.4260]	78.39% [68.35%−86.02%]
80 and older	0.3546 [0.3032–0.4072]	0.7961 [0.6109–1.0118]	1.0910 [0.8124–1.4387]	207.75 [167.9%−253.3%]

**Table 3:** Projected number of non-Hispanic Black adults 50 years and older with chronic disease (in millions by sex and age) in the United States.

**Age cohort**	**2020**	**2035**	**2050**	**Relative change between 2020 and 2050 (%)**
≥**1 chronic disease (million)**				
50–59 years	1.8920 [1.7732–1.9764]	2.0731 [1.9372–2.1709]	2.5934 [2.4242–2.7151]	37.07% [36.71%−37.37%]
60–79 years	5.1999 [4.9250–5.4070]	7.9304 [7.2875–8.4762]	9.2501 [8.5027–9.8901]	77.89% [72.64%−82.91%]
80 and older	1.1075 [0.9373–1.2359]	2.4979 [1.8941–3.0597]	3.3778 [2.4076–4.3753]	205.0% [156.9%−254.0%]
Total	8.1994 [7.6355–8.6193]	12.5014 [11.118–13.706]	15.2213 [13.33–16.98]	85.64% [74.64%−97.01%]
**Female**				
50–59 years	0.9467 [0.8744–0.9975]	1.0329 [0.9505–1.0909]	1.2791 [1.1773–1.3507]	35.11% [34.65%−35.41%]
60–79 years	2.7028 [2.5303–2.8349]	4.1817 [3.8298–4.4699]	4.8336 [4.4260–5.1707]	78.84% [74.92%−82.39%]
80 and older	0.6737 [0.5631–0.07625]	1.5029 [1,1131–1.8587]	2.0498 [1.4143–2.6938]	204.3% [151.2%−253.3%]
**Male**				
50–59 years	0.9453 [0.8974–0.9870]	1.0402 [0.9848–1.0890]	1.3143 [1.2445–1.3755]	39.03% [38.68%−39.37%]
60–79 years	2.4972 [2.3780–2.5996]	3.7487 [3.4577–4.0099]	4.4165 [4.0767–4.7237]	76.86% [71.44%−81.71%]
80 and older	0.4337 [0.3742–0.4853]	0.9949 [0.7809–1.2010]	1.3280 [0.9934–1.6815]	206.2% [165.5%−246.5%]
**Multimorbidity (million)**				
50–59 years	0.2130 [0.1681–0.2580]	0.2338 [0.1843–0.2848]	0.2929 [0.2307–0.3565]	37.48% [37.27%−38.16%]
60–79 years	0.6167 [0.4735–0.7695]	0.9357 [0.6438–1.2800]	1.0918 [0.7531–1.4922]	77.05% [59.05%−93.91%]
80 and older	0.1328 [0.0878–0.1890]	0.2765 [0.1597–0.4568]	0.3658 [0.1960–0.6403]	175.5% [123.1%−238.9%]
Total	0.9625 [0.7294–1.2165]	1.446 [0.9878–2.0216]	1.7505 [1.1798–2.489]	81.87% [61.75%−104.6%]
**Female**				
50–59 years	0.0972 [0.0737–0.1241]	0.1064 [0.0805–0.1365]	0.1318 [0.0997–0.1691]	35.53% [35.18%−36.26%]
60–79 years	0.2963 [0.2135–0.3879]	0.4604 [0.2880–0.6765]	0.5319 [0.3329–0.7824]	79.53% [55.93%−101.6%]
80 and older	0.0778 [0.0506–0.1117]	0.1622 [0.0862–0.2635]	0.2171 [0.1065–0.3743]	178.9% [110.5%−234.9%]
**Male**				
50–59 years	0.1158 [0.0944–0.1375]	0.1274 [0.1038–0.1517]	0.1611 [0.1311–0.1919]	39.13% [38.905–39.57%]
60–79 years	0.3204 [0.2523–0.3943]	0.4753 [0.3457–0.6236]	0.5599 [0.4082–0.7334]	74.75% [61.83%−86.00%]
80 and older	0.0550 [0.0372–0.0778]	0.1144 [0.0692–0.1933]	0.1487 [0.0851–0.2667]	170.6% [128.7%−243.1%]

**Table 4:** Projected number of Hispanic adults 50 years and older with chronic disease (in millions by sex and age) in the United States.

**Age cohort**	**2020**	**2035**	**2050**	**Relative change between 2020 and 2050 (%)**
≥**1 chronic disease (million)**				
50–59 years	2.5907 [2.4400–2.7267]	2.9035 [2.7270–3.0625]	3.6682 [3.4463–3.8678]	41.59% [41.24%−41.85%]
60–79 years	7.3341 [7.0190–7.6569]	11.835 [11.153–12.659]	14.056 [13.239–15.044]	91.66% [88.62%−96.49%]
80 and older	1.8748 [1.6667–2.1631]	4.7988 [3.9698–6.1682]	7.0086 [5.5290–9.7020]	273.8% [231.7%−348.5%]
Total	11.7996 [11.125–12.546]	19.537 [17.849–21.889]	24.732 [22.214–28.613]	109.61% [99.67%−128.1%]
**Female**				
50–59 years	1.2915 [1.2059–1.3694]	1.4372 [1.3377–1.5272]	1.7908 [1.6673–1.9025]	38.66% [38.26%−38.93%]
60–79 years	3.7812 [3.6062–3.9682]	6.1549 [5.7926–6.5917]	7.2247 [6.7941–7.7456]	91.07% [88.40%−95.19%]
80 and older	1.1254 [0.9961–1.3028]	2.8186 [2.3239–3.6155]	4.1383 [3.2314–5.7171]	267.7% [224.4%−338.8%]
**Male**				
50–59 years	1.2992 [1.2295–1.3581]	1.4663 [1.3837–1.5361]	1.8774 [1.7722–1.9664]	44.51% [44.14%−44.79%]
60–79 years	3.5529 [3.4094–3.7141]	5.6809 [5.3608–6.0676]	6.8316 [6.4450–7.2994]	92.28% [89.03%−96.53%]
80 and older	0.7494 [0.6629–0.8603]	1.9802 [1.6114–2.5527]	2.8703 [2.2253–3.9849]	283.0% [235.7%−363.2%]
**Multimorbidity (million)**				
50–59 years	0.3028 [0.2302–0.3902]	0.3381 [0.2555–0.4365]	0.4278 [0.3235–0.5528]	41.27% [40.53%−41.68%]
60–79 years	0.9176 [0.6792–1.1838]	1.4275 [0.9545–1.9999]	1.6943 [1.1353–2.3721]	84.65% [67.15%−100.3%]
80 and older	0.2428 [0.1619–0.3280]	0.5578 [0.3114–0.8892]	0.7915 [0.4188–1.3337]	225.9% [158.6%−306.7%]
Total	1.4632 [1.0713–1.902]	2.3234 [1.5214–3.3156]	2.9136 [1.8776–4.2586]	99.13% [75.26%−123.9%]
**Female**				
50–59 years	0.1371 [0.0973–0.1837]	0.1522 [0.1074–0.2045]	0.1897 [0.1337–0.2551]	38.31% [37.40–38.87%]
60–79 years	0.4331 [0.2953–0.5854]	0.6847 [0.4105–0.9842]	0.8024 [0.4811–1.1539]	85.24% [62.94%−97.13%]
80 and older	0.1389 [0.0864–0.1937]	0.3143 [0.1603–0.5314]	0.4495 [0.2165–0.8224]	223.7% [150.6%−324.5%]
**Male**				
50–59 years	0.1657 [0.1329–0.2096]	0.1859 [0.1482–0.2356]	0.2381 [0.1898–0.3021]	43.73% [42.83%−44.15%]
60–79 years	0.4844 [0.3747–0.6135]	0.7428 [0.5270–1.0157]	0.8919 [0.6336–1.2181]	84.12% [69.12%−98.55%]
80 and older	0.1040 [0.0753–0.1359]	0.2436 [0.1511–0.3677]	0.3420 [0.2014–0.5492]	228.9% [167.4%−304.2%]

**Table 5:** Projected number of other adults 50 years and older with chronic disease (in millions by sex and age) in the United States.

**Age cohort**	**2020**	**2035**	**2050**	**Relative change between 2020 and 2050 (%)**
≥**1 chronic disease (million)**				
50–59 years	1.0999 [0.9828–1.1846]	1.2283 [1.0907–1.3279]	1.5519 [1.3788–1.6769]	41.10% [40.29%−41.56%]
60–79 years	3.0872 [2.8448–3.3042]	4.8468 [4.2676–5.3310]	5.7521 [5.0644–6.3334]	86.32% [78.02%−91.68%]
80 and older	0.7201 [0.5662–0.8631]	1.7029 [1.1205–2.3582]	2.3800 [1.4159–3.6244]	230.5% [150.1%−319.9%]
Total	4.9072 [4.3938–5.3519]	7.778 [6.4788–9.0171]	9.684 [7.8591–11.6347]	97.34% [78.87%−117.4%]
**Female**				
50–59 years	0.5487 [0.4836–0.5946]	0.6086 [0.5328–0.6619]	0.7583 [0.6642–0.8245]	38.19% [37.33%−38.66%]
60–79 years	1.5952 [1.4542–1.7136]	2.5305 [2.2494–2.7866]	2.9676 [2.6363–3.2725]	86.03% [81.28%−90.97%]
80 and older	0.4340 [0.3396–0.5224]	1.0081 [0.6614–1.3750]	1.4154 [0.8355–2.1206]	226.1% [146.1%−305.9%]
**Male**				
50–59 years	0.5511 [0.4992–0.5923]	0.6198 [0.5579–0.6688]	0.7936 [0.7146–0.8560]	43.99% [43.16%−44.51%]
60–79 years	1.4920 [1.3693–1.5906]	2.3163 [2.0182–2.5507]	2.7844 [2.4282–3.0656]	86.63% [77.33%−92.73%]
80 and older	0.2861 [0.2266–0.3423]	0.6948 [0.4591–0.9844]	0.9646 [0.5804–1.5037]	237.2% [156.2%−339.3%]
**Multimorbidity (million)**				
50–59 years	0.1103 [0.0614–0.1523]	0.1231 [0.0693–0.1695]	0.1555 [0.0871–0.2146]	40.98% [41.78%−40.89%]
60–79 years	0.3176 [0.1315–0.4941]	0.4458 [0.1377–0.8072]	0.5289 [0.1653–0.9555]	66.51% [2.74%−93.39%]
80 and older	0.0840 [0.0304–0.1709]	0.1584 [0.0335–0.4794]	0.2083 [0.0369–0.7504]	148.0% [21.5%−339.1%]
Total	0.5119 [0.2233–0.8173]	0.7273 [0.2405–1.4561]	0.8927 [0.2893–1.9205]	74.39% [29.56%−134.9%]
**Female**				
50–59 years	0.0498 [0.0270–0.0723]	0.0552 [0.0301–0.0800]	0.0688 [0.0373–0.0999]	38.01% [38.28%−38.12%]
60–79 years	0.1493 [0.0611–0.2508]	0.2125 [0.0635–0.4316]	0.2487 [0.0749–0.5054]	66.63% [22.70%−101.5%]
80 and older	0.0478 [0.0172–0.0957]	0.0890 [0.0185–0.2656]	0.1175 [0.0201–0.4220]	145.9% [16.7%−341.1%]
**Male**				
50–59 years	0.0605 [0.0345–0.0807]	0.0678 [0.0392–0.0910]	0.0867 [0.0498–0.1165]	43.42% [44.52%−44.43%]
60–79 years	0.1683 [0.0704–0.2506]	0.2332 [0.0742–0.4081]	0.2801 [0.0904–0.4886]	66.41% [28.38%−94.96%]
80 and older	0.0362 [0.0132–0.0752]	0.0695 [0.0150–0.2137]	0.0908 [0.0168–0.3283]	150.8% [27.8%−336.5%]

The projected number of non-Hispanic Blacks with at least one chronic condition is 8.1994 million (7.6355–8.6193) in 2020 and is expected to increase to 15.2213 million (13.33–16.98) by 2050 [that is a relative change between 2020 and 2050 of 85.64% (74.64–97.01)]. Most non-Hispanic Blacks with one chronic condition and multimorbidity are females between 60 to 79 years old. Similarly, to all the races, the age group with the highest increase is individuals 80 years and older for both one chronic condition and multimorbidity. The number of non-Hispanic Blacks with multimorbidity is projected to increase from 0.9625 million (0.7294–1.2165) in 2020 to 1.7505 million (1.1798–2.489) by 2050, representing a relative increase of 82.87% (61.75%−104.6%).

Hispanic adults 50 years and older with at least one chronic condition are estimated to increase from 11.7996 million (11.125–12.546) in 2020 to 24.732 million (22.214–28.613) by 2050. This change represents an increase of 109.61% (99.67–128.1). Like all races, most Hispanics with one chronic condition are females within the age group of 60 to 79 years, and the age group with the highest increase in the number of people with at least one chronic condition and multimorbidity is individuals aged 80 years and older. Also, the number of Hispanics with multimorbidity is projected to increase from 1.4632 million (1.0713–1.902) in 2020 to 2.9136 million (1.8776–4.2586) by 2050. Most Hispanics with multimorbidity are males between 60 to 79 years old.

Lastly, the number of other races who are not non-Hispanic Whites, Blacks, or Hispanics in the United States with at least one chronic condition is projected to increase from 4.9072 million (4.3938–5.3519) in 2020 to 9.684 million (7.8591–11.6347) by 2050, representing a relative increase of 97.34% (78.87–117.4) from 2020 to 2050. Most of the other races with one chronic condition are females between the ages of 60–79 years. Among the other race, the age group with the highest increase in the number of people with at least one chronic condition and multimorbidity is individuals aged 80 years and older. The projected number of adults 50 years and older categorized as other races with multimorbidity is estimated to increase from 0.5119 million (0.2233–0.8173) in 2020 to 0.8927 million (0.2893–1.9205) by 2050. Most of the other races with multimorbidity are females.

## Discussion

The results show that the number of people in the United States aged 50 years and older is projected to increase significantly. Consequently, by 2050, most individuals 50 years and older will have one or more chronic conditions. Most of the population 50 years and older with one or more chronic conditions are projected to be between the ages of 60 to 79 years, and the number of individuals 80 years and older with one or more chronic conditions is expected to more than double from 2020 to 2050. Most individuals 50 years and older with one chronic condition are females, while that with multimorbidity are males.

The insight that the majority of the adult population 50 years and older, across all races, will have at least one chronic condition has health and economic implications. Within the health domain, these insights emphasize the importance of prioritizing the promotion of access to high-quality primary care services that can provide whole-person care that ensures prevention and management of chronic disease care and address evidence-based social determinants of health that increase the risk of developing chronic diseases. Moreover, individual, family and community-oriented health education that highlights the importance of a healthy lifestyle and addresses structural issues that perpetuate health disparities should be a vital part of the health system to change the trajectory of chronic disease. The health education provided to individuals, families and the community and care models offered to the population should emphasize the continuous care models for addressing chronic conditions that help individuals to lead better lives. This health education and care models should focus on self-care (i.e., tasks performed by healthy people to stay healthy) and self-management (i.e., day-to-day tasks undertaken to reduce the impact of chronic disease on physical health status) approaches. These approaches should focus on encouraging the individual to stay healthy and for those with chronic conditions, the ability to manage the symptoms, treatment, physical and psychosocial consequences, and lifestyle changes inherent in living with a chronic condition.

Chronic disease and especially multimorbidity, is associated with increased mortality (6), reduced quality of life, and functional status (2, 7, 8), increased health services use (3, 9), and higher cost of care. As a result, health care systems and policymakers should prioritize cost-effective interventions that have the potential to reduce the cost of chronic disease management to the health care system. Chronic disease is associated with substantial work productivity losses. Thus, policymakers and employers should focus on programs and resource allocation to reduce the incidence and prevalence of chronic disease and absenteeism resulting from chronic diseases to maintain and increase productivity.

The main strength of this paper is the use of 20 years' worth of data to estimate the incidence and prevalence of chronic diseases among the adult population in the United States. The main limitation of this research is, first, the list of chronic diseases included in the Health and Retirement Study is not a comprehensive list of chronic diseases, and the chronic diseases reported in the survey are self-reported. A broader definition of chronic diseases would include more conditions that are not captured in this study. These can potentially underestimate the incidence and prevalence of chronic diseases projected in this study. Hence, the numbers provided in the research should be interpreted within the context of the chronic diseases captured in the survey used herein. Another important limitation is that individuals transitioning to the adult population are assumed to have similar chronic disease transition patterns observed in the Health and Retirement Survey. Lastly, a limitation of the statistic model is that since the data used for this study (Health and Retirement Study) is not an annual survey, we fill in gaps with pseudo-data representing successive years to obtain annual transition probabilities.

## Data availability statement

Publicly available datasets were analyzed in this study. This data can be found at: The Health and Retirement Study.

## Author contributions

JA conceived and designed the study, developed the multi-state population model to simulate the chronic disease burden among the adult population in the USA, and conducted the analysis and manuscript writing. C-TC conducted the statistical analysis for the transition probabilities using the Health and Retirement Study and developed the R algorithm used for data analytics. All authors contributed to the article and approved the submitted version.

## References

[1] Barnett K Mercer SW Norbury M Watt G Wyke S Guthrie B. Epidemiology of multimorbidity and implications for health care, research, and medical education: a cross-sectional study. Lancet. (2012) 380:37–43. 10.1016/S0140-6736(12)60240-2 22579043

[2] van den Akker M Buntinx F Metsemakers JF Roos S Knottnerus JA. Multimorbidity in general practice: prevalence, incidence, and determinants of co-occurring chronic and recurrent diseases. J Clin Epidemiol. (1998) 51:367–75. 10.1016/S0895-4356(97)00306-5 9619963

[3] Walker AE. Multiple chronic diseases and quality of life: patterns emerging from a large national sample, Australia. Chronic Illn. (2007) 3:202–18. 10.1177/1742395307081504 18083677

[4] Wolff JL Starfield B Anderson G. Prevalence, expenditures, and complications of multiple chronic conditions in the elderly. Arch Intern Med. (2002) 162:2269–76. 10.1001/archinte.162.20.2269 12418941

[5] Salisbury C Johnson L Purdy S Valderas JM Montgomery AA. Epidemiology and impact of multimorbidity in primary care: a retrospective cohort study. Br J Gen Pract. (2011) 61:e12–21. 10.3399/bjgp11X548929 21401985 PMC3020068

[6] Fortin M Lapointe L Hudon C Vanasse A Ntetu AL Maltais D. Multimorbidity and quality of life in primary care: a systematic review. Health Qual Life Outcomes. (2004) 2:51. 10.1186/1477-7525-2-51 15380021 PMC526383

[7] Kadam UT Croft PR North Staffordshire GP Consortium Group. Clinical multimorbidity and physical function in older adults: a record and health status linkage study in general practice. Fam Pract. (2007) 24:412–9. 10.1093/fampra/cmm049 17698977

[8] Gijsen R Hoeymans N Schellevis FG Ruwaard D Satariano WA van den Bos GA. Causes and consequences of comorbidity: a review. J Clin Epidemiol. (2001) 54:661–74. 10.1016/S0895-4356(00)00363-2 11438406

[9] Nguyen H Manolova G Daskalopoulou C Vitoratou S Prince M Prina AM. Prevalence of multimorbidity in community settings: a systematic review and meta-analysis of observational studies. J Comorb. (2019) 9:2235042X19870934. 10.1177/2235042X19870934 31489279 PMC6710708

[10] Bodenheimer T Sinsky C. From triple to quadruple aim: care of the patient requires care of the provider. Ann Fam Med. (2014) 12:573–6. 10.1370/afm.1713 25384822 PMC4226781

[11] Arnetz BB Goetz CM Arnetz JE Sudan S vanSchagen J Piersma K. Enhancing healthcare efficiency to achieve the Quadruple Aim: an exploratory study. BMC Res Notes. (2020) 13:362. 10.1186/s13104-020-05199-8 32736639 PMC7393915

[12] Schoen R. Practical uses of multistate population models. Annu Rev Sociol. (1988) 14:341–61. 10.1146/annurev.so.14.080188.002013 12315442

[13] Ansah JP Koh V Chiu C-T Chei C-L Zeng Y Yin Z-X. Projecting the number of elderly with cognitive impairment in china using a multi-state dynamic population model. Syst Dyn Rev. (2017) 33:89–111. 10.1002/sdr.158134157986

[14] Ansah JP Inn RLH Ahmad S. An evaluation of the impact of aggressive hypertension, diabetes, and smoking cessation management on CVD outcomes at the population level: a dynamic simulation analysis. BMC Public Health. (2019) 19:1105. 10.1186/s12889-019-7429-2 31412830 PMC6694535

[15] Ahmad SC. Evaluating the impact of upstream and downstream interventions on chronic kidney disease and dialysis care: a simulation analysis. Syst Dyn Rev. (2021) 37:32–58. 10.1002/sdr.1676

[16] Arias E. United States life tables, 2010. Natl Vital Stat Rep. (2014) 63:1–63. Available online at: https://www.cdc.gov/nchs/data/nvsr/nvsr63/nvsr63_07.pdf 25383611

[17] Fisher GG Ryan LH. Overview of the health and retirement study and introduction to the special issue. Work Aging Retire. (2018) 4:1–9. 10.1093/workar/wax032 29423243 PMC5798643

[18] Cai L Hayward MD Saito Y Lubitz J Hagedorn A Crimmins E. Estimation of multi-state life table functions and their variability from complex survey data using the SPACE Program. Demogr Res. (2010) 22:129–58. 10.4054/DemRes.2010.22.6 20463842 PMC2867357

[19] Matchar DB Ansah JP Koh V Whitson HE. Trajectories of functional ability over the life course: a conceptual model of the interaction of stressor-induced functional loss and resilience. Syst Dyn Rev. (2018) 34:481–502. 10.1002/sdr.1611 33041497 PMC7544242

[20] Urwannachotima N Hanvoravongchai P Ansah JP Prasertsom P Koh VRY. Impact of sugar-sweetened beverage tax on dental caries: a simulation analysis. BMC Oral Health. (2020) 20:76. 10.1186/s12903-020-1061-5 32183817 PMC7079374

[21] Sterman JD. System dynamics modeling: tools for learning in a complex world. Calif Manage Rev. (2001) 43:8–25. 10.2307/41166098

